# The myocutaneous gastrocnemius flap for periprosthetic joint infection of the knee

**DOI:** 10.1002/jeo2.12089

**Published:** 2024-07-06

**Authors:** Seraina L. C. Müller, Richard Kuehl, Dirk J. Schaefer, Mario Morgenstern, Martin Clauss, Rik Osinga

**Affiliations:** ^1^ Department of Plastic Reconstructive, Aesthetic and Hand Surgery, University Hospital Basel Basel Switzerland; ^2^ Center for Musculoskeletal Infections (ZMSI) University Hospital Basel Basel Switzerland; ^3^ Infectious Diseases and Hospital Epidemiology University Hospital Basel Basel Switzerland; ^4^ Department of Orthopaedic and Trauma Surgery University Hospital Basel Basel Switzerland; ^5^ Canniesburn Plastic Surgery Unit Glasgow Royal Infirmary Glasgow UK; ^6^ Praxis beim Merian Iselin Basel Switzerland; ^7^ REHAB Basel, Clinic for Neurorehabilitation and Paraplegiology Basel Switzerland

**Keywords:** flap, gastrocnemius flap, infection, knee, orthoplastic, orthoplastic surgery, periprosthetic joint infection, soft‐tissue reconstruction, sural artery perforator

## Abstract

**Purpose:**

Periprosthetic joint infection (PJI) following total knee arthroplasty (TKA) presents significant challenges, especially in elderly and comorbid patients, often necessitating revision surgeries. We report on a series of patients with confirmed PJI of the knee and concomitant soft‐tissue/extensor apparatus defects, treated by using pedicled myocutaneous medial or lateral sural artery perforator (MSAP/LSAP) gastrocnemius flaps.

**Methods:**

Our retrospective study at the Center for Musculoskeletal Infections, included patients with knee PJI undergoing pedicled myocutaneous MSAP/LSAP gastrocnemius flap reconstruction for combined soft tissue and extensor apparatus defects. The tendinous back of the gastrocnemius muscle was used and, if required, the Achilles tendon for extensor apparatus reconstruction, with the skin island addressing the cutaneous defect. Perioperative complications and postoperative outcomes after 1 year were evaluated, including functional and clinical assessments with the American Knee Society Score (AKSS).

**Results:**

Eight patients (mean age 73 years; five female) were included, predominantly with Staphylococcus aureus infections. Six patients involved isolated MSAP flaps, two were extended with the Achilles tendon. The median time for wound healing was 9 days. Short‐term follow‐up showed successful reconstruction in seven patients, with minor wound dehiscence in one patient. One patient required flap revision for a perigenicular haemato‐seroma and two patients were diagnosed with new haematogenous PJI infection. Significant improvement in AKSS scores after surgery was observed (functional AKSS: median 33–85; clinical AKSS: median 64–91, *p* = 0.001).

**Conclusion:**

Pedicled myocutaneous MSAP/LSAP gastrocnemius flaps offer a safe, reliable and versatile option for reconstructing combined soft tissue and extensor apparatus defects in PJI after TKA. This approach yields excellent functional outcomes with minimal peri‐ and postoperative complications, which is particularly beneficial in elderly and comorbid patients and feasible in settings without microsurgical availability.

**Level of evidence:**

Level IV.

**Abbreviations**
AKSS: American Knee Society Score
ASA: American Society of Anesthesiologists
DAIR: debridement, antibiotics and implant retention
F: female
IQR: interquartile range
LSAP: lateral sural artery perforator
M: male
MSAP: medial sural artery perforator
PJI: periprosthetic joint infection
TKA: total knee arthroplasty

## INTRODUCTION

The incidence of periprosthetic joint infection (PJI) after total knee arthroplasty (TKA) is increasing globally [1]. The growing number of elderly and more comorbid patients undergoing primary arthroplasty implies a greater number of revision surgeries and higher rates of PJI. The comorbidities of patients contribute to host‐related risk factors, aggravating the potential for complications such as PJI with concomitant soft tissue defects. Infection and subsequent radical debridement create a defect that can affect not only the soft tissue envelope, but also the underlying extensor apparatus of the knee. Soft tissue reconstruction is crucial for adequate treatment of PJI to avoid loss of function and therefore prevent arthrodesis of the knee or amputation. PJI presents a significant challenge for orthoplastic reconstruction, and a multidisciplinary strategy is mandatory.

The myocutaneous medial and lateral sural artery perforator (MSAP/LSAP) gastrocnemius flap represents a relatively new flap design for soft tissue reconstruction around the knee [5, 6, 7]. Following the principle to reconstruct ‘like with like’, the tendinous back of the muscle can be used to reconstruct the extensor apparatus/capsular defect of the knee [15]. If needed, the tendinous portion of the flap can be extended with the Achilles tendon to reconstruct a complete rupture of the quadriceps or patellar tendon [2]. Implantation of nonresorbable foreign body material can thereby be avoided, which is beneficial in the treatment of PJI. The skin island of the flap can reconstruct the cutaneous defect. The gastrocnemius muscle provides an extra layer of well‐perfused tissue to (i) serve as a barrier against new infection and (ii) provide a transport vehicle to deliver nutrients and the antibiotic agent to the site of infection.

The objective of this retrospective, single centre study was to report the clinical outcome after reconstruction of combined soft tissue and extensor apparatus defects in PJI of the knee with a pedicled myocutaneous MSAP/LSAP gastrocnemius flap.

## PATIENTS AND METHODS

All patients with confirmed PJI have been recorded in a prospectively maintained database since 2019. We retrospectively searched (2019–2022) this database for patients with PJI after TKA and concomitant perigenicular soft tissue/extensor apparatus defects who were reconstructed with either a pedicled MSAP or LSAP myocutaneous gastrocnemius flap. PJI was defined according to its European Bone and Joint Infection Society 2021 definition [12]. It was treated with debridement and implant retention (DAIR), one‐stage or two‐stage exchange, according to the algorithm published by Zimmerli et al. [20]. The extensor apparatus defect was either preoperatively known (complete rupture) or intraoperatively confirmed (partial rupture).

The patient‐specific demographic data included sex, date of birth, age at operation, disease‐associated comorbidities and body mass index. Approval was obtained from the local ethics committee (EKNZ 2019‐00265). Data on surgical treatment included the number of surgical procedures before soft tissue reconstruction with the MSAP/LSAP myocutaneous gastrocnemius flap and direct closure of the donor site. Short‐term follow‐up (<3 weeks) included perioperative complications according to the Clavien‐Dindo classification [4], time to complete wound healing and cumulative time of in‐patient stay. Short‐term follow‐up was determined based on previous findings involving a larger patient cohort with similar settings, which indicated that flap‐related complications typically occur within the first 3 weeks after surgery [13]. Major flap complications were defined as partial or total flap loss requiring revision surgery. Minor flap complications were defined as flap necrosis or prolonged wound secretion not needing surgical revision. Uneventful wound healing was regarded as flap donor site and flap inset healing without dehiscence and with stable soft tissues at a maximum of 3 weeks after surgery [13]. The minimal time for follow‐up was defined as 12 months after definitive PJI surgery, according to the Delphi‐based international multidisciplinary consensus guidelines [3].

The clinical and functional evaluation of the knee was assessed with the American Knee Society Score (AKSS) before and after surgery at last follow‐up [8]. The aesthetic result of the flap was rated (bad, satisfying and good) by another, not involved independent surgeon.

### Statistical analysis

The data were analysed by using the Jamovi project (2022, Version 2.3). All variables were evaluated for normal distribution with a combination of histograms and Shapiro–Wilk tests. Continuous variables are presented as means with standard deviations (SDs) and minimum and maximum range when following the Gaussian distribution. For skewed data, the median, interquartile range (IQR) and minimum and maximum range was used. Friedman's test was applied to compare the AKSS at the date of hospital admission and last follow‐up.

## RESULTS

In total, 108 patients were treated for PJI between 2019 and 2022, of which eight patients (7%) were included in this study (five male, three female) with a mean age of 73 (SD 13) years. The demographics and comorbidities of the patients are outlined in Table 1. Six patients had a monomicrobial PJI, one patient had a polymicrobial infection and one patient was culture negative.

**Table 1:** Patient data and surgical, infection and follow‐up details.

	Patient no.							
	1	2	3	4	5	6	7	8
Sex/age	F/88	F/71	F/85	M/60	M/76	F/48	F/78	M/78
ASA	2	3	3	2	3	3	3	3
Pathogen	*Staphylococcus aureus*	No pathogen identified (culture negative)	*Enterobacter faecalis*	*Staphylococcus epidermidis*	*S. aureus*	*Enterobacter cloacae*	No pathogen identified (culture negative)	*S. epidermidis*
PJI treatment	Two‐stage	DAIR	Two‐stage	Two‐stage	Two‐stage	One‐stage	Two‐stage	Two‐stage
Flap type	Pedicled MSAP gastrocnemius with Achilles tendon	Pedicled LSAP gastrocnemius	Pedicled LSAP gastrocnemius	Pedicled MSAP gastrocnemius with Achilles tendon	Pedicled MSAP gastrocnemius	Pedicled MSAP gastrocnemius	Pedicled MSAP gastrocnemius	Pedicled MSAP gastrocnemius
Antibiotics	Rifampicin + Ciprofloxacin	Co‐amoxicillin	Vancomycin, Ertapenem, Daptomycin	Co‐amoxicillin, Vancomycin, Cefepime, Rifampicin, Clindamycin	Co‐amoxicillin, Flucloxacillin, Levofloxacin, Cefazolin, Daptomycin, Rifampicin, Ciprofloxacin	Co‐amoxicillin, Cefepime, Ciprofloxacin	Cefazolin	Co‐amoxicillin, Ertapenem, Vancomycin
Duration of antibiotics (days)	90	10	47	90	134	97	7	42
Surgery time (min)	283	165	290	316	240	234	240	270
Clavien‐Dindo grade	0	0	0	1	2	2	0	0
Time to heal (days)	5	35	18	17	10	5	7	7
Hospital stay (days)	8	17	20	21	32	8	11	18
Complications		Partial flap necrosis, small wound healing disorder		New haematogenous PJI			Seroma	New haematogenous PJI
Follow‐up time (days)	837	817	1097	691	369	832	606	372
Aesthetic result	Good	Satisfying	Good	Good	Good	Good	Satisfying	Good
Pain	No	No	No	No	No	No	No	No
Strength/Knee flexion	M5/110°	M5/110°	M5/120°	M4/120°	M5/100°	M5/90°	M5/90°	M4/100°
Knee extension lag	0°	0°	0°	20°	10°	15°	0°	5°
AKSS clinical/functional at infection	67/50	97/50	69/20	49/25	55/60	46/40	85/20	60/60
AKSS clinical/functional at follow‐up	97/80	99/100	99/30	89/90	85/90	73/90	93/70	85/75

Abbreviations: AKSS, American Knee Society Score; ASA, American Society of Anesthesiologists; DAIR, debridement and implant retention; F, female; LSAP, lateral sural artery perforator; M, male; M4, strength meaning muscle contraction that overcomes some resistance; M5, strength grade meaning normal muscle strength, MSAP, medial sural artery perforator; PJI, periprosthetic joint infection.

### Surgical procedure and PJI treatment

**Figure 1:**
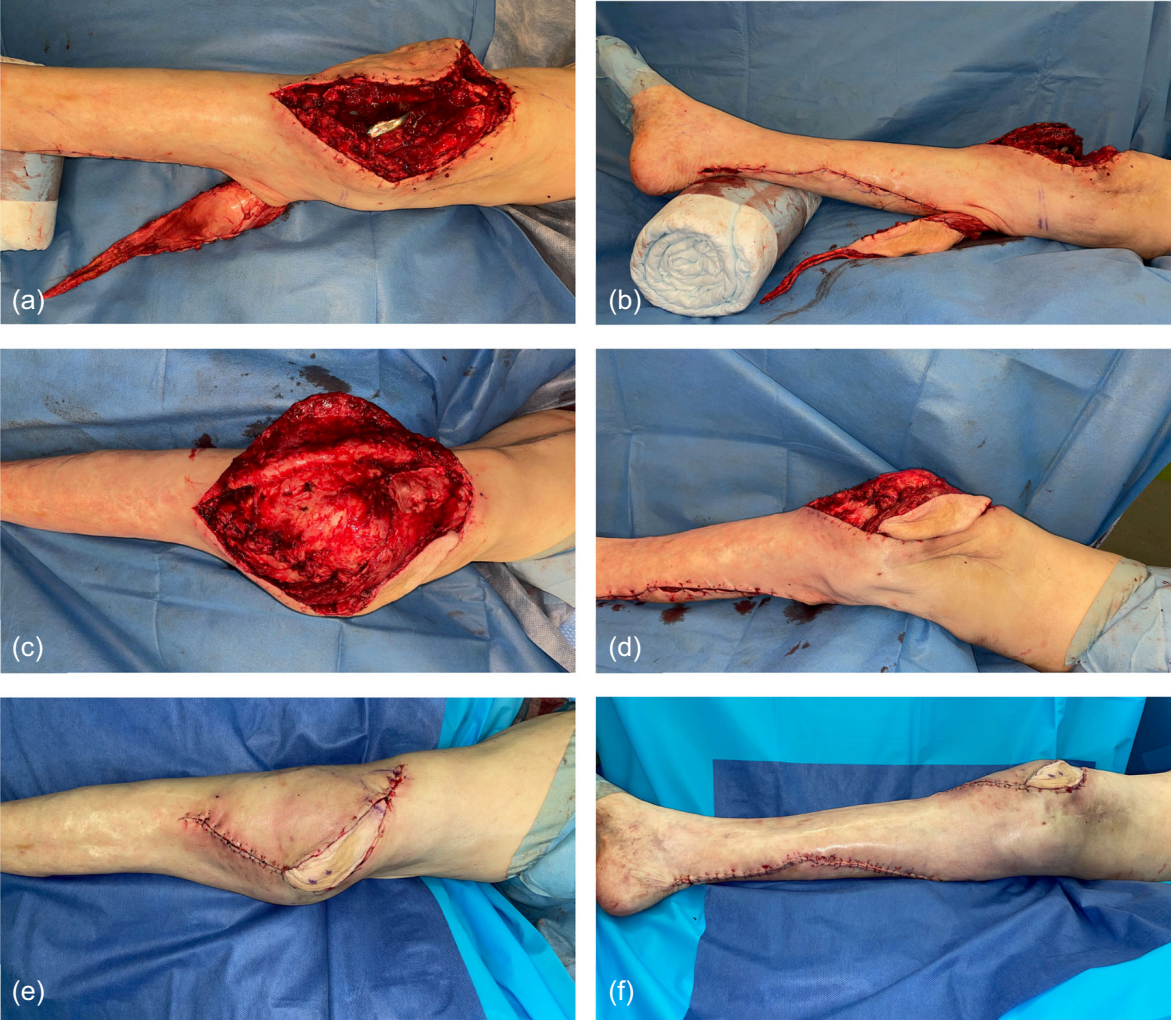
Intraoperative view of the ruptured extensor apparatus (quadriceps tendon) of a 76‐year‐old patient (a, b). The myocutaneous medial sural artery perforator (MSAP) gastrocnemius flap is raised with one‐third of the Achilles tendon (extended flap raise) and the donor site is provisionally closed. The pedicled flap has been put in place through a subcutaneous tunnel, preserving the greater saphenous vein and the saphenous nerve (c, d). Note how the tendinous back of the medial gastrocnemius muscle (a) is used to reconstruct the ruptured extensor apparatus according to the principle to reconstruct ‘like with like’. According to the same principle, the (MSAP) skin island is used to reconstruct the cutaneous defect resulting from (i) the muscle bulk of the gastrocnemius flap and (ii) positioning of the flap when reconstructing the extensor apparatus with the knee in a bent position. Anterior (e) and medial view (f) at the end of the operation. The skin island of the flap is well perfused (the two blue dots mark the two perforators) and the donor site could be closed directly. The knee is left in full extension for 8 weeks due to the complete rupture of the quadriceps tendon.

**Figure 2:**
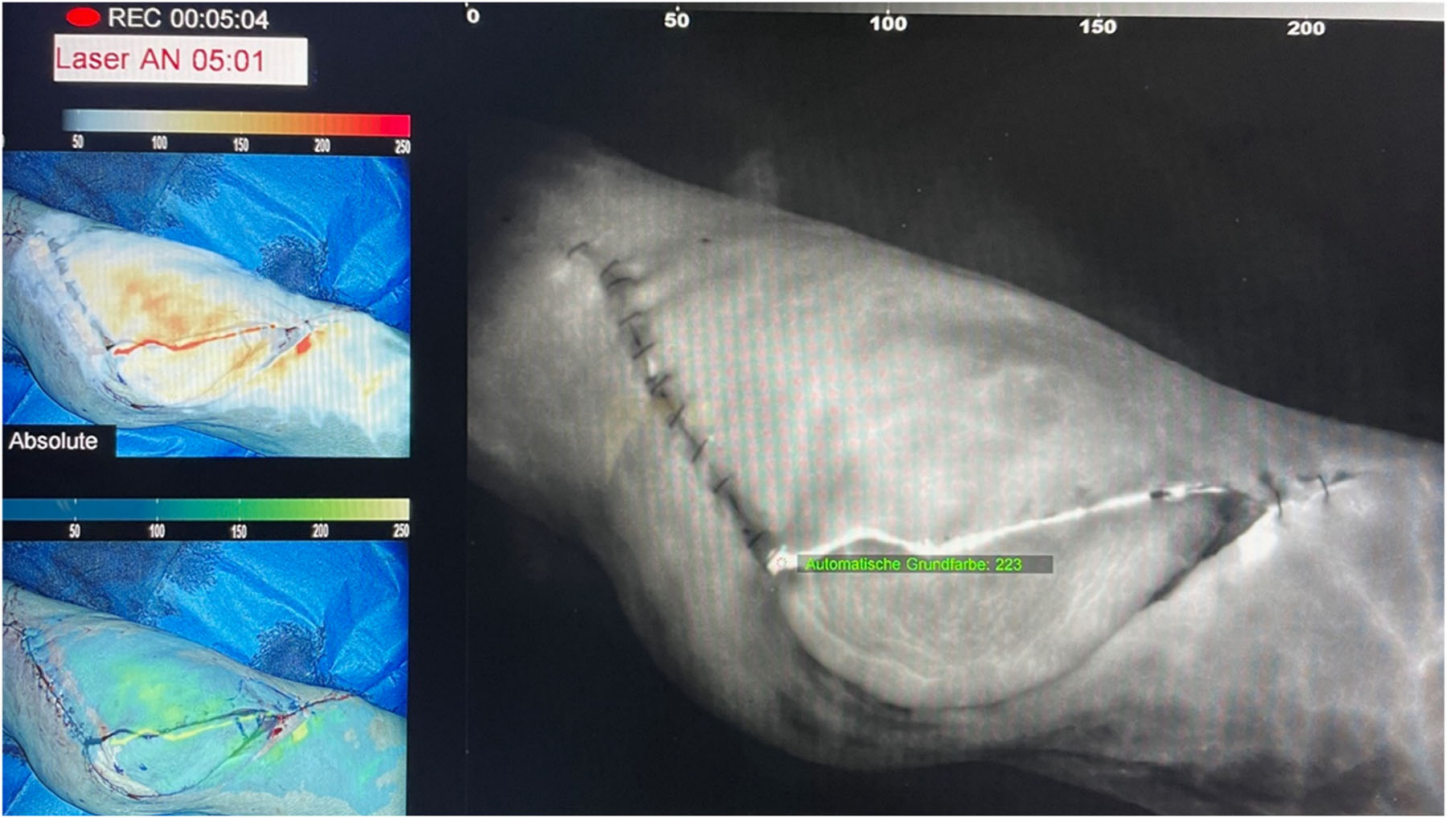
Intraoperative blood flow measurement of the soft tissues of both the local and the flap tissue with indocyanine green.

Six patients involved isolated MSAP flaps, two were extended with the Achilles tendon. A one‐stage procedure was performed in two patients (1x DAIR, 1x one‐stage exchange) and a two‐stage procedure in six patients with a spacer implantation at the first stage. In all patients, soft tissue reconstruction was performed at the first stage. Six patients were treated with an MSAP myocutaneous gastrocnemius flap and two with an LSAP myocutaneous gastrocnemius flap, depending on the localisation of the soft tissue defect. The tendinous dorsum of the flap was used to reconstruct the extensor apparatus (quadriceps or patellar tendon/knee capsula). The skin island of the flap was designed in such a way that (i) it could reconstruct the soft‐tissue defect, (ii) it included the fasciocutaneous perforator and (iii) all donor sites could be closed primarily [17] (Figures 1 and 2). The mean duration of the entire orthoplastic (orthopaedic and reconstructive) operation was 255 (SD 45) min. All patients received antibiotic treatment postoperatively according to a well‐established algorithm [20]. Patients with a partial extensor apparatus defects were immobilised in bed for 5 postoperative days with the knee in extension. The knee joint was then mobilised depending on the soft‐tissue conditions, generally increasing flexion 20° per week. In the two patients with a complete rupture of the extensor apparatus the knee joint was left in full extension for a total time of 8 weeks.

### Postoperative course

The mean cumulative in‐patient stay was 17 (SD 8) days. According to the Clavien‐Dindo classification, one patient had a grade I complication (pain and wound healing disorder) and two patients had a grade II complication (anaemia). No higher graded complications were observed. There was no flap loss and no minor flap complications. One postoperative wound healing disorder was treated conservatively. After a median of 9 (IQR: 11, 5–35) days, all wounds were stable and without dehiscence. Late complications were observed in three patients. One patient developed a haemato‐seroma at the donor site that was evacuated surgically, and two patients were diagnosed with new haematogenous PJI of the knee 352 and 599 days after PJI revision (new infection with different bacteria compared with initial PJI), which were both successfully treated with a DAIR procedure that included flap elevation. All patients completed at least 12 months of follow‐up calculated from the day of the last surgery (mean 25, SD 8, range 12–36 months).

### American Knee Society Score (AKSS)

Preoperatively, patients presented with a median clinical score of 64 out of 100 and a functional score of 33 out of 100. During the last follow‐up, a median clinical score of 91 out of 100 and a functional score of 85 out of 100 were measured. Friedman's test showed a significant improvement in both the clinical (*p* = 0.001) and the functional score (*p* = 0.001).

The mean active range of motion of the knee at the last follow‐up was 105 degrees (90–120 degrees), with an active extensor lag of 10 degrees in two patients and 20 degrees in one patient. In two patients, strength was diminished (graded M4), whereas in the other six patients, it was normal (graded M5). One patient had moderate and occasional pain when walking. Walking distance was limited to 400 m in one patient, 400–800 m in a second patient and unlimited in all other patients.

### Infection control

Two patients developed a new PJI during follow‐up (Table 1). Patient No. 4 had a new infection 2 years after flap procedure with a new pathogen due to an acute haematogenous infection with *Staphylococcus lugdunensis*. Therefore, a DAIR procedure with successful flap elevation was performed. At the last follow‐up, the patient had no infection, was pain free and could walk without walking aids for long distances despite a slight loss of strength (M4). Patient No. 8 had undergone multiple surgeries due to PJI with *S. epidermidis* before orthoplastic reconstruction. He was 12 months free of infection when a new haematogenous infection with *S. aureus* occurred and a successful DAIR procedure was performed. At last follow‐up, the patient was pain free and without signs of persistent PJI.

## DISCUSSION

The orthopaedic treatment algorithm for PJI of the knee is well‐known [9, 20]. Intact soft tissues, whether original tissue or the result of plastic‐surgical reconstruction, are a prerequisite for successful PJI treatment.

As presented here, patients with PJI of the knee can have a concomitant soft tissue defect, which can involve the extensor apparatus and the knee capsula [15]. In these patients, a combined multidisciplinary approach is particularly important and they should be treated in a specialised bone and joint infection unit to ensure the best possible outcome [9, 19]. The interaction between various specialists as part of the orthoplastic treatment concept allows a simultaneous, multidisciplinary approach while the patient is located in one institution.

A soft tissue envelope around the knee that does not allow tension‐free direct closure is an indication for plastic‐surgical reconstruction. This need must be anticipated early and therefore patients should be referred early. In general, combined soft tissue defects around the knee can be reconstructed with local, pedicled or free flaps. These flaps consist either of fasciocutaneous tissue alone, muscular tissue alone (often with a split thickness skin graft) or a combination of tissues (composite, fascio‐musculocutaneous flaps). Local flaps are based on local perforating vessels, which in an area of previous surgery and infection are not a safe option. The medial and lateral gastrocnemius muscle can serve as a pedicled flap. These flaps are well‐known workhorses for soft tissue defects around the knee due to their reliability, comparative ease of harvest and low donor site morbidity [10, 14], which is supported by our series. Free flaps can be an option if the defect is anticipated to be of such size and extent (extensor apparatus) that a pedicled flap would be insufficient for reconstruction.

The choice of flap depends both on the preoperative analysis of the soft tissue defect and on the individual patient's characteristics [10, 15]. In general, soft tissue reconstruction must be stable, durable and functional. Therefore, we advocate following the reconstructive principle to replace ‘like with like’. Thus, the tendinous back of the gastrocnemius muscle was used to reconstruct the capsular/extensor apparatus defect and the perforator‐based skin island to reconstruct the cutaneous defect, which enlarges because of the muscle bulk of the gastrocnemius muscle during flap inset. Depending on the localisation of the composite defect, either a myocutaneous MSAP or LSAP gastrocnemius flap was used. Of note, the musculocutaneous perforators over the medial gastrocnemius muscle appear to be more reliable than when used over the lateral gastrocnemius muscle [11, 18]. Nevertheless, a recent study presented a series of 27 patients undergoing orthoplastic reconstruction with a pedicled, myocutaneous lateral gastrocnemius flap in which the authors emphasised the reliability of the flap [16]. Regarding the surgical complexity (free flap surgery), we advocate going as complex as necessary and balancing this with the patients' comorbidities and functional needs. Here, excellent functional results could be reported with pedicled flaps.

In all patients with a two‐stage procedure, soft tissue reconstruction was performed during the first stage. The rationale for this was fourfold: First, early surgery maximises the time for the soft tissue to heal and integrate. Second, the flap tissue acts as a vehicle to transport nutrients and antimicrobial agents to the site of infection. Third, the smaller the number of interventions, the lower the rate of complications and the smaller the number of anaesthetic interventions. Fourth, the myocutaneous gastrocnemius flap can be reraised without complications at the second stage, as demonstrated.

This study has limitations, such as its retrospective design. This may have caused bias during data collection and analysis. Furthermore, the relatively small number of patients treated limits the generalisation of the results. The absence of a comparative cohort treated with an alternative therapeutic modality precludes definitive conclusions regarding treatment efficacy. And lastly, the minimal follow‐up period of one year in our study does restrict our understanding of long‐term results, as these would need a minimal follow‐up time of more than five years.

## CONCLUSIONS

The pedicled myocutaneous MSAP/LSAP gastrocnemius flap provides a safe, reliable, and versatile option for reconstructing soft tissue and extensor apparatus defects in PJI after TKA. This approach delivers excellent functional early results with minimal peri‐ and postoperative complications, which is particularly advantageous in elderly and comorbid patients.

## AUTHOR CONTRIBUTIONS

All listed authors contributed substantially to this work (Seraina L. C. Müller, Martin Clauss and Rik Osinga for the study conception and design; Seraina L. C. Müller for the data collection, the data analysis; Seraina L. C. Müller, Martin Clauss, Rik Osinga for the data interpretation; Seraina L. C. Müller, Richard Kuehl, Mario Morgenstern, Dirk J. Schaefer, Martin Clauss, Rik Osinga for the drafting of the manuscript, the figures, and the literature search) and approved the submission to KSSTA.

## CONFLICT OF INTEREST STATEMENT

The authors declare no conflict of interest.

## ETHICS STATEMENT

Approval was obtained from the local ethics committee of Switzerland (EKNZ 2019‐00265).

## Data Availability

The data sets used and/or analysed during the current study are available from the corresponding author upon reasonable request.
